# Chemopreventive effect of 4′-hydroxychalcone on intestinal tumorigenesis in Apc^Min^ mice

**DOI:** 10.3892/ol.2021.12741

**Published:** 2021-04-20

**Authors:** Qing Chen, Jiahong Lei, Jinzhe Zhou, Shaoze Ma, Qi Huang, Bujun Ge

Oncol Lett 21: Article no. 213, 2021; DOI: 10.3892/ol.2021.12474

Subsequently to the publication of the above article, the authors have realized that, in Fig. 1, the chemical structure of 4’-hydroxychalcone was presented without one of its requisite double bonds.

A corrected version of Fig. 1, including the correct chemical structure of 4’-hydroxychalcone, is shown below. The authors are grateful to the Editor of *Oncology Letters* for granting them the opportunity to publish this corrigendum, and regret any inconvenience caused to the readership of the Journal.

## Figures and Tables

**Figure 1.**
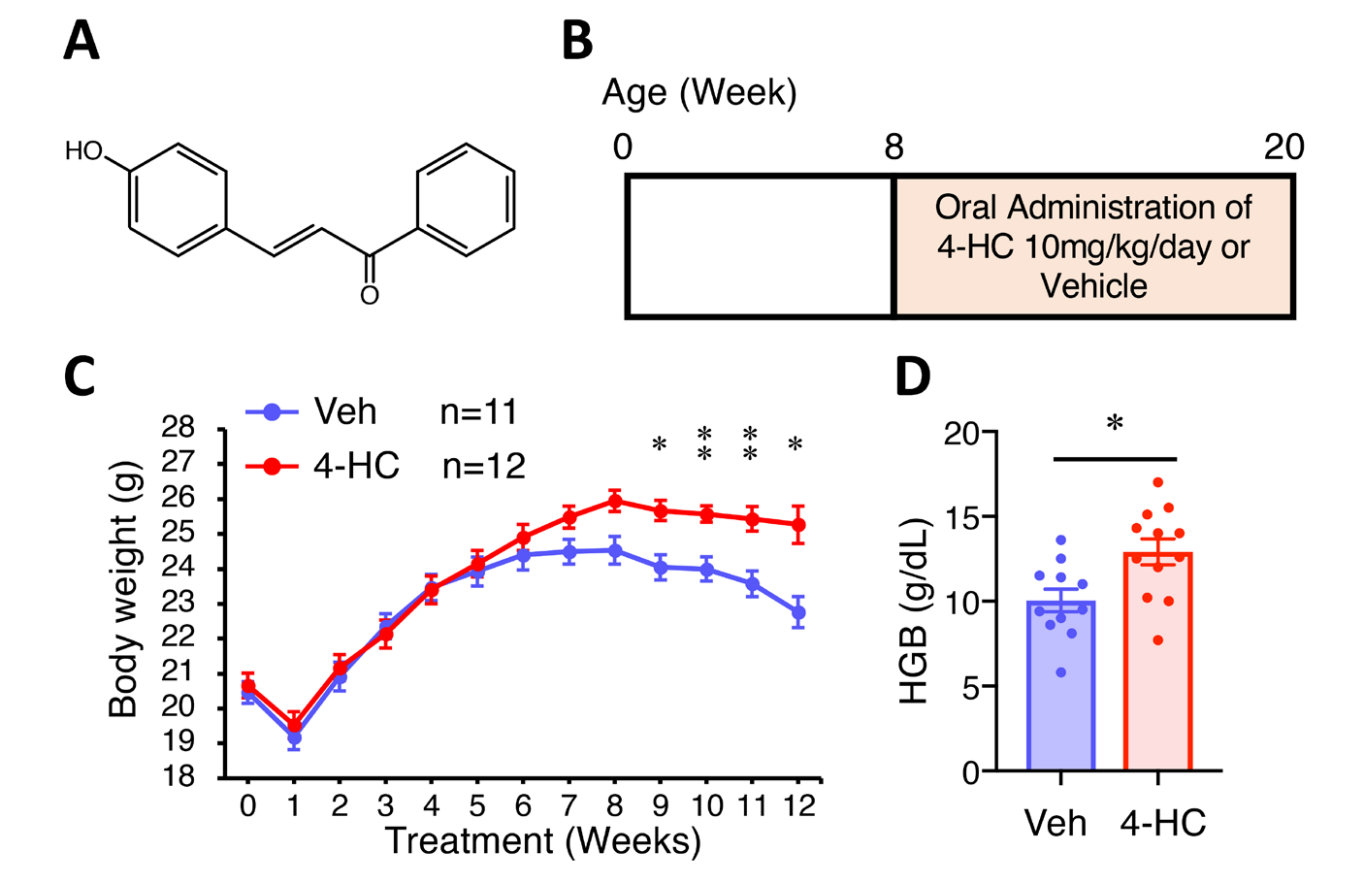
Experimental design and general *in vivo* observations. (A) The chemical structure of 4-HC. (B) Experimental design for the evaluation of the chemopreventive effect of 4-HC in male ApcMin mice. Mice were randomly allocated into groups for oral administration with 10 mg/kg/day 4-HC (n=12) or Veh control (n=11) from 8 to 20 weeks of age. Changes in (C) body weight over time and (D) HBG levels at 20 weeks for ApcMin mice orally treated with 4-HC (n=12) or Veh (n=11). Data are presented as the mean ± SEM. *P<0.05, **P<0.01 vs. Veh. 4-HC, 4’-hydroxychalcone; ApcMin, adenomatous polyposis coli multiple intestinal neoplasia mouse model; HGB, hemoglobin; Veh, vehicle.

